# The impact of smoking on oral health and patient assessment of tobacco cessation support from Turkish dentists

**DOI:** 10.18332/tid/136418

**Published:** 2021-06-10

**Authors:** Arzu Beklen, Burak G. Yildirim, Mehmet Mimaroglu, Muhammet B. Yavuz

**Affiliations:** 1 Department of Periodontology, Faculty of Dentistry, Eskisehir Osmangazi University, Eskisehir, Turkey

**Keywords:** dentist, tobacco cessation, public health, oral health, Turkey

## Abstract

**INTRODUCTION:**

Dentists are in a critical position to help patients quit smoking. This study analyses the effectiveness of Turkish dentists in smoking cessation as part of routine patient care.

**METHODS:**

An in-person cross-sectional survey on previous dental visit experiences was completed by 226 patients recruited from the Department of Periodontology, Eskisehir Osmangazi University, Turkey, from March 2019 to September 2019. The questionnaire included topics on patient’s smoking/quit characteristics, experiences on smoking cessation from their dentists, and willingness for the implementation of smoking cessation advice by dentists.

**RESULTS:**

In all, 38% of the patients were current smokers, 8% were former smokers, and 68% tried to quit previously. Smokers demonstrated consistently higher scores for plaque index, gingival index, and probing depth, than former/non-smokers (p<0.05). Patients’ knowledge of adverse effects was high, and the patients presented a positive attitude toward receiving cessation activities from dentists (86.7%). A total of 89% responded positively to be asked about their smoking behavior. However, the dentists’ approach for cessation discussions did not go any further than listing the harmful effects. Only 32% of the patients were informed about side effects of smoking and one-third were encouraged to quit. In general, offering smoking cessation advice was relatively infrequent, and the majority of patients tried to quit smoking by themselves (76%) without using any nicotine replacement product (84%).

**CONCLUSIONS:**

Smoking leads to oral health problems. Dentists in Turkey may ask their patients’ about their smoking habits but less frequently offer practical help to quit.

## INTRODUCTION

Cigarette smoking causes more than 8 million deaths per year worldwide. Estimates, announced in previous years, anticipated more millions of deaths from diseases attributable to smoking by 2030^1,2^. Therefore, prevention and cessation of smoking should be amongst the most important priorities of healthcare systems. Smoking is associated with several different adverse effects, several of which have a clear impact on oral health, such as periodontal diseases^3^.

Raising questions about social concerns like stained teeth and bad breath sometimes can be effective reasons to stop smoking. Whatever the motivation to start the cessation program, healthcare providers have the highest impact on smokers to motivate them to stop smoking, and the cessation program requires repeated interventions by healthcare providers and several attempts by patients^4^. In all, 40.4% of the adult Turkish population reported that they visited a dentist within the previous year^5^, and due to education policies, monitoring the oral health of patients has been increasing continuously. Furthermore, in recent years Turkey became one of the popular destinations for health tourism, of which dental tourism is a leading area in this sector. Therefore, dentists have a unique place to guide smoking cessation advice to their patients on different occasions.

Nicotine, the ingredient of tobacco, leads to addiction which requires patient-specific multi-step guidance to escape from chronic dependence^6^. Dental treatment needs to be carried out over several sessions^7^; therefore, the dentists are in a perfect position to provide up-to-date cessation information with long-term success during the repeated dental appointments. However, despite the awareness to involve dental professionals successfully, dentists lack the competence to undertake smoking cessation in patients^8^.

Addictions other than smoking, such as alcohol, can be a serious topic for dentists to discuss, and patients may not feel comfortable having discussions on that topic^9^. Therefore, understanding the barriers from the point of both dentists and patients will be more interesting and productive compared to fighting against smoking^10^, since asking, advising and referring patients to a counsellor or quitline would take only a few minutes^11^.

In behavioral approaches to cessation, the environment provides cues to action and consequences of an action to influence the choices of individuals. Whatever the psychological reasons for smokers or whatever the situations they are in, stopping smoking becomes a more considered action, which is affected by those around smokers^12^. At this point, it is certain that dentists are in a critical position to reflect on all health aspects and improve patients’ behavior. However, it brings up another concern such as do the dental patients want to seek help to quit smoking from their oral health care providers? Overall, most of the literature studied the course of action from the point of the dentists. However, a limited number of studies have analyzed the patient’s expectations on the attitude of dentists. To our knowledge, this is the first study to measure the patients’ demand in dental practitioners’ clinics in Turkey. We aimed to analyze the opinion of patients toward receiving information from the dentists and to assess their previous dentists’ motivation in smoking cessation activities.

## METHODS

This cross-sectional survey was conducted at Eskisehir Osmangazi University, in the Department of Periodontology, through random sampling among patients from March 2019 to September 2019. The questions were asked to patients at the time of their first dental visit before having any dental treatment or counselling within the department. The questions of the survey referred to the experience of previous dental visits in the past. There was no time limit to fill in the questionnaire before starting the dental examination in the clinic. After data were collected through the questionnaire, the clinical evaluation of periodontal parameters was performed.

### Patient questionnaire

The survey was conducted among dental patients in the periodontology clinic for dental trainees under the supervision of a specializing dentist for each patient (n=226). After the patients were welcomed to the dental unit, information was given by a dentist, and their consent was obtained to fill the survey. Patients were aged ≥18 years and did not have any language or reading barrier. The Eskisehir Osmangazi University, Ethics Committee approved the study (#25403353– 050.99–E.26275). The reliability of the survey was measured for internal consistency using Cronbach’s alpha test. A Cronbach value of 0.70 was considered an acceptable measure of reliability^13^. The questionnaire comprised 33 questions under these topics: 1) basic demographic data; 2) oral hygiene habits; 3) dental visit frequency; 4) secondhand exposure, awareness about the systemic consequences of smoking; 5) previous dentists’ interest about their smoking; and 6) patients’ expectation about dentists’ interest for smoking.

If the patients were smokers, the following additional topics were covered: 1) type of smoking behavior; 2) awareness about the oral consequences of smoking; 3) use of other tobacco products; 4) previous experiences to quit; 5/6) previous discussion/training in the dental settings about side effects and cessation protocols; 7) demand to receive quit advice from a dentist; 8) feelings having a discussion on smoking; and 9) readiness to quit smoking with the help of a dentist.

For the questions in which specific responses are not possible, e.g. ‘If your dentist helps you to quit smoking, would you consider stopping smoking?’, response items were measured on a 5-point Likert scale ranging from ‘strongly agree’ to ‘strongly disagree’. So as not to influence the responses of patients, the examination findings in the smoking group were stated after the completion of the questionnaire.

### Periodontal parameters

After completing the survey, the patients’ clinical periodontal parameters were assessed to determine their oral health status. The plaque index^14^ was recorded by moving the probe along the gingival margin (0=no plaque, 1=plaque on probe, 2=visible plaque by the naked eye, and 3=abundance of soft matter). The gingival index^15^, was recorded 20 seconds after moving the periodontal probe along the gingival sulcus of a tooth (0=no bleeding, 1=isolated bleeding spots visible, 2=blood forms a confluent red line along the margin, and 3=heavy or profuse bleeding). The probing depth^16^ was determined by measuring the distance from a gingival margin to the base of the sulcus.

### Statistical analysis

The collected data were recorded into MS Excel 2003 and exported to SPSS Statistical Software version 21.0 (Armonk, NY: IBM Corp.). Descriptive statistics were generated on patient sociodemographic and all other variables. Chi-squared tests were used for the comparison of categorical data. The results were assessed at a 95% confidence interval and at a significance level of 0.05.

## RESULTS

### Patients’ characteristics

The demographics of 226 patients who accepted to take the survey are given in Table 1. A total of 44.7% of respondents had graduated from university, and 34.5% of respondents did not have a monthly income (Table 1).

**Table 1:** Description of the study sample at Eskisehir Osmangazi University, Turkey, March–September 2019 (N=226)

*Characteristics*	*n*	*%*
**Sex**		
Male	90	39.8
Female	136	60.2
**Age** (years)		
18–28	76	33.6
29–39	56	24.8
40–50	52	23.0
51–61	27	11.9
≥61	15	5.8
**Education level**		
Primary school	41	18.1
Middle school	20	8.8
High school	53	23.5
University	101	44.7
Postgraduate	11	4.7
**Income** (TRY)		
Unemployed	78	34.5
1800–2000	33	14.6
2001–2500	30	13.3
2501–3500	30	13.3
3501–5000	29	12.8
≥5000	26	11.5

TRY: 1000 Turkish Lira about 120 US$.

### Periodontal parameters

There were statistically significant differences between the smokers, former smokers and nonsmokers with regard to their periodontal parameters. Higher plaque amounts were found in the smokers (2.78±0.92) compared to non-smokers (1.0±0.6) and former smokers (1.1±0.8). GI was examined to assess the severity of inflammation, which presented clear severity in smoking groups. The GI for smokers was 2.5±0.5, for non-smokers it was 0.5±0.4, and for former smokers 1.9±1.0. The observation of PD revealed that the current smokers had a higher mean probing depth (5.6±1.9) than non-smokers (1.6±0.8) and former smokers (2.4±1.3) (p<0.05).

### Patients’ knowledge on negative effects of smoking and oral health maintenance

A majority of the patients (84.5%) showed awareness of the negative risks of smoking on oral tissues. In all, 80.3% reported that oral cavity health affects systemic health. Patients’ knowledge that oral health affects systemic health varied according to education level (p<0.05), and increased with the higher level of school education obtained.

The majority of the patients (82.2%) brush their teeth a minimum once a day, 40% two times a day, and 13.8% more than two times. Regarding brushing times, 36.4% of patients reported to brush their teeth for 30–60 seconds, whereas 16.4% up to 90 seconds, 18.7% up to 120 seconds, and 13.8% brush for more than two minutes. In contrast, 83.2% of patients do not use dental flossing. Of all patients, 44.7% reported that regular dental check-ups must be done every 6 months, whereas 11.1% reported at 3-month intervals, and 10.6% reported once a year.

### Patients’ characteristics on smoking

In all, 53.5% of responders (n=121) have never smoked, 38.0% (n=86) were current smokers with 15.2±10.9 cigarettes/day and 8.4% (n=19) of patients were former smokers who have smoked at least 100 cigarettes in their lifetime (18.6±13.0 cigarettes/day). Of current smokers, 68.4% reported failing to quit, 75% of the patients prefer not to smoke frequently in the mornings, and 73.7% do not find it difficult to refrain from smoking in places where it is forbidden. With regard to other nicotine dependence questions, 65.3% of smokers do not smoke if they are sick, and 17% smoke within 5 minutes after waking. With regard to dual use, 90.4% of patients do not use any other tobacco products other than conventional cigarettes (others: electronic cigarette 4.3%, water pipe 3.2%, cuban cigar 1.1%, pipe 1.1%); 57.1% of responders live with a smoker in the house and only 15.9% of the patients have five close friends who do not smoke.

### Patients’ previous experience in quitting smoking

**Figure 1:**
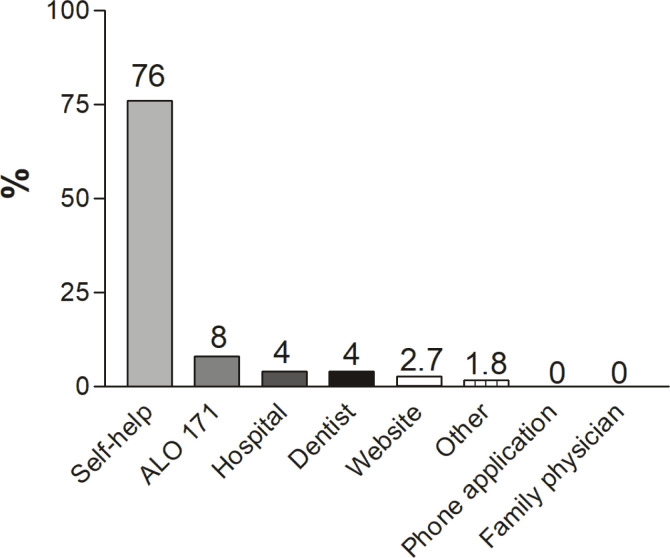
Patients’ previous cessation supports. ALO 171: Free Cessation Call Center of the Ministry of Health

**Figure 2:**
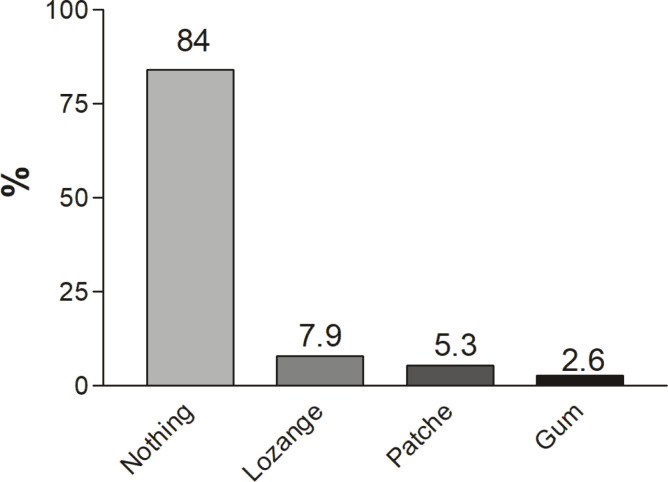
Used nicotine replacement products during patients’ previous cessation strategies

In all, 68.4% of smokers had tried to quit previously. Most of the patients tried to quit smoking by themselves (76%). Of importance, ALO-171, which is the free quitline of the Turkish Ministry of Health, was called by only 8% of respondents (Figure 1), and only 15.8% used nicotine replacement (Figure 2).

### Previous dental visit experiences on patients’ tobacco history evaluation and consultation activities along with patients’ expectations

Of the previous dentists of the patients, 31.6% mentioned the adverse effects of smoking, and 32.7% of those patients were warned by their dentists to quit smoking. However, 89.4% of the patients are willing to be asked about their smoking behavior by their dentists (7.5% do not want, 3.1% have no idea), and 86.7% want to be instructed to quit by their dentists. Similarly, a total of 86.7% responded positively (30.6% strongly angry, 56.1% agree) to quitting via counselling provided by a dentist and 8.2% of respondents had no idea, 5.1% strongly disagreed. Patients’ attitude for positive decisions about quitting upon counselling by a dentist was agreed by 48.4% of respondents (9.5% strongly agree, 38.9% agree, 28.4% had no idea, 18.9% disagree, and 4.2% strongly disagree). Furthermore, to understand the feelings on a question about daily smoking amount, only 2.1% ‘agreed’. Otherwise, 41.2% of respondents feel ashamed, and 39.2% had no negative feelings being asked about their daily smoking amounts. Significant differences in patients’ preference to be asked regarding smoking behavior by a dentist are observed according to age (18–24 years, 1st youngest group out of 5) (p<0.05), gender (male) (p<0.05) and income (4th highest out of 5) (p<0.05).

## DISCUSSION

Smoking has detrimental effects on oral tissues. In terms of periodontal health, smoking clearly increases the presence and severity of periodontal disease. Dental patients are aware of the negative effects of smoking on their oral health and want to be instructed by dentists to quit smoking. However, Turkish dentists are more active in asking about smoking habits with their patients than in offering practical support for cessation. Deeper pockets, greater probing depths, more attachment loss are the expected negative influences of smoking in the clinical parameters of periodontal health. Due to the penetration of the smoking derived-cytotoxic substances, the tissues lead to the immune response, which results in exacerbated tissue destruction^3^. This clearly suggests that smoking has a direct influence on periodontal tissues when compared to non-smokers and former smokers.

Dentists are the health providers who can see the adverse effects attributable to tobacco smoking immediately. However, despite the possible easy recognition opportunity, most dental providers do not offer smoking cessation advice^8^. A short discussion between 3–10 minutes with a healthcare provider raises the success in smoking cessation 1.6-fold, and if the discussion continues more than 10 minutes, the likelihood reaches 2.3 fold^17^. Unfortunately, although our patients were performing good oral hygiene and ready to hear conversations about smoking cessation, the oral healthcare providers did not help to quit. It is obvious that the dialogue between dentists and patients does not go far beyond side-effect warning in Turkey.

In the brain, the specific receptors are triggered by nicotine, and these receptors have a customary nicotine level. Whenever that level drops, the brain starts to look for nicotine^18^. It is of importance patients attempt to quit on their own without using any additional strategies, such as nicotine replacement, which causes them to go back to smoking because of the withdrawal symptoms^19^. In fact, smokers usually are not aware of the reason for physiological craving activity in the brain. They are even not aware that the more support they have to quit, the better their chances of quitting. Unfortunately, most of our patients did not receive any replacement therapy or help, which in turn failed with a high percentage. It is interesting to see that most of the patients did not call the free hotline ALO 171, which is supported by The Turkish Ministry of Health, to offer free advice and medical treatment to those trying to quit smoking. It seems that with the lack of direct healthcare provider assistance, the odds of a smoker’s success with cessation decreases under all circumstances.

Behavioral support with pharmacotherapy is considered the most effective way to quit smoking^17^. It is thus a critical because the physicians have the possibility to support integrating a variety of technology tools in patients’ quitting attempts, including the cessation applications via smartphones^20^. It was a unique observation that most of our patients were surprised when we asked if they used a smartphone app to quit smoking. Unfortunately, none of our patients quit smoking through a phone application because they did not know that such an application existed. However, many of our patients said that they would download the application if available.

In practice, smokers usually need many attempts, sometimes as many as ten or more, before they are able to quit^21^. The multi-step process of dental settings can provide an encouragement to move forward the efforts to quit smoking^22^. It is important to remember that other smokers also exist around our patients, like family members and friends. Dentist’s and patient’s face-to-face unique discussions will contribute to indirect information propagation for society as well^23^. Furthermore, light and intermittent smoking youth are equally as likely to either quit smoking or become heavier smokers^24^. In the clinics handling mostly young patients, the dentists can be one of the main influencers in the social environment to prevent the onset of smoking in adolescence^23^.

Therefore, the reasons for the dentists not to take advantage of this situation must be considered carefully. Several factors should be considered in interpreting the reasons for this gap between the expectations of patients and the quitting service provided by dentists. Earlier studies indicated that dentists ask their patients about smoking, whereas only fewer than 10% of dental providers assist with a quit attempt^25^. On the contrary, our study indicates that the patients are ready to listen to the advice of dentists. This discrepancy could be explained along with various obstacles. First, the dentists could lack time^26^. This study was conducted in a university clinic, in which treatment expenses are covered mostly by the government. The majority of our patients reported low income and most probably preferred similar free public dental services earlier. Naturally, providing free treatment always causes an increase in the number of patients applying for public services and limits the time allocated per patient. Second, no matter whether the patient was treated in a public or a private clinic, the lack of reimbursement for counseling is another barrier in providing tobacco cessation^27^. Maybe, the more realistic reason than all mentioned is the dentists, and individual practices need to agree on the roles of dentists in smoking cessation^28^.

Dentists are in the perfect position to play a role in promoting healthy lifestyles by incorporating tobacco cessation programs into their daily practices. Actually, the role of dentists in encouraging their patients to stop smoking had not been considered as an essential opportunity until the last 15–20 years^29^. In recent years, only a few countries have developed anti-smoking opportunities for dentists to use during routine dental check-ups^22^. However, despite encouragements to involve dentists to help patients to quit smoking, only a few dentists included interventions against smoking as part of routine check-ups^30^. Although various reasons, such as lack of patient education materials or lack of knowledge of available referral resources, are presented by dentists^29,31^, training for dental staff and compensation of their time must be first addressed, to ensure the implementation of a smoking cessation program in Turkey.

Turkey is fighting against smoking with radical changes in the rules, such as the extension of banning smoking in private cars^32^. In addition to these rules, efforts to regulate the coverage of smoking cessation intervention would be evidence-based patient care in Turkey’s dental healthcare system^33^. Based on the literature, the mainly used strategies to quit smoking can be summarized as behavioral therapy, nicotine replacement therapy, and pharmacological therapy^34^. In this regard, the study of Houston et al.^35^, which carried out an approach via email for dental patients who are smokers, is an example of a motivational tool for behavioral therapy to quit smoking in daily practice^35^. Informing patients to receive further advice from their dentists encourages them to stop smoking and provides an opportunity to the repeated interventions by healthcare providers to stop the smoking habit. It is noteworthy that, in daily dental practice, it is suggested dentists limit themselves to brief interventions and counselling sessions to light smoker patients. In contrast, the dentists must refer heavy smokers or those with serious addiction problems to specialists or psychologists^36^. Overall, the method, which will be used in the interventions, must depend on the level of addiction and must be planned according to patient-specific factors. In Turkey, the implementation of a class-based counseling teaching^37^ will direct dentists toward patient-specific therapies as the long-term strategy.

On several occasions, Turkey highlighted the importance of oral health and the health consequences of smoking on media with the support of the government^38^. Almost all patients are aware of the harmful effects of smoking and the importance of good oral hygiene with some requirements to improve. Despite the many deficiencies in quitting smoking, it appears that in the future, if dentists start to talk with their patients about quitting, already pre-informed patients will be more likely to succeed in quitting smoking.

### Strengths and limitations

The study consisted of a wide range of people who lived throughout the city of Eskisehir. Eskisehir is not only an intersection point of major cities but also known as a university city, which makes our survey representative of a wide spectrum of patients’ points of view. Furthermore, compared to electronic surveys, we obtained higher response rates by using an in-person interview-type survey, and respondents did not show any unwillingness to use time on the survey^39,40^. These were clear strengths of the present study since almost all patients who were invited to participate in the study accepted to fill in the questionnaire. However, respondents who considered themselves under the focus of dentists were likely to become over-represented. The reality of smoking cessation readiness by dentists may thus be lower than our results suggest. While the short-term impact of this study shows that dental patients are highly motivated to be instructed by their dentists, the long-term effectiveness of dentists in helping their patients to quit smoking cannot be known. This limitation of the study is worthy of note. Further research that evaluates longer follow-up would be valuable, especially when combined with a certain number of strategies with repeated interventions by dentists. In this first report for the Turkish population, it is clear that dentists do not offer support to their patients, whereas patients are ready to be instructed. This may lead to the conclusion that less time is needed to succeed in helping patients to quit smoking in the dental setting. This focus shows that investment and policy changes are needed in increasing the role of the dentist in promoting cessation within dental settings in Turkey. To identify a certain solution for the above concerns, we recommend further research, specifically on how to educate dental students to have adequate information on smoking cessation support.

## CONCLUSIONS

From our point, dentists are critical in identifying the negative effects of smoking on periodontal tissues and must incorporate programs for patients to help them quit smoking. Turkish patients consider smoking a major health problem and want to be instructed by their dentists for smoking cessation, yet they seldom receive practical cessation support. Increased awareness of the roles of dentists in the overall smoking cessation and prevention activities is needed in the dental healthcare setting of Turkey.

## Data Availability

The data supporting this research cannot be made available for privacy reasons.
